# Trinity of inflammation, innate immune cells and cross-talk of signalling pathways in tumour microenvironment

**DOI:** 10.3389/fphar.2023.1255727

**Published:** 2023-08-17

**Authors:** Ali Attiq, Sheryar Afzal

**Affiliations:** ^1^ Discipline of Pharmacology, School of Pharmaceutical Sciences, Universiti Sains Malaysia, Minden, Penang, Malaysia; ^2^ Department of Biomedical Sciences, Faculty of Veterinary Medicine, King Faisal University, Al Ahsa, Saudi Arabia

**Keywords:** innate immunity, inflammation, signalling pathways, cross-talks, tumorigenesis, tumour microenvironment, tumour-promoting inflammation

## Abstract

Unresolved inflammation is a pathological consequence of persistent inflammatory stimulus and perturbation in regulatory mechanisms. It increases the risk of tumour development and orchestrates all stages of tumorigenesis in selected organs. In certain cancers, inflammatory processes create the appropriate conditions for neoplastic transformation. While in other types, oncogenic changes pave the way for an inflammatory microenvironment that leads to tumour development. Of interest, hallmarks of tumour-promoting and cancer-associated inflammation are striking similar, sharing a complex network of stromal (fibroblasts and vascular cells) and inflammatory immune cells that collectively form the tumour microenvironment (TME). The cross-talks of signalling pathways initially developed to support homeostasis, change their role, and promote atypical proliferation, survival, angiogenesis, and subversion of adaptive immunity in TME. These transcriptional and regulatory pathways invariably contribute to cancer-promoting inflammation in chronic inflammatory disorders and foster “smouldering” inflammation in the microenvironment of various tumour types. Besides identifying common target sites of numerous cancer types, signalling programs and their cross-talks governing immune cells’ plasticity and functional diversity can be used to develop new fate-mapping and lineage-tracing mechanisms. Here, we review the vital molecular mechanisms and pathways that establish the connection between inflammation and tumour development, progression, and metastasis. We also discussed the cross-talks between signalling pathways and devised strategies focusing on these interaction mechanisms to harness synthetic lethal drug combinations for targeted cancer therapy.

## 1 Introduction

Inflammation is an evolutionary process that allows the human body to cope with pathogenic invasion and traumatic injuries. It is involved in the activation and recruitment of numerous innate and adaptive immune cells that bolter the host’s defence against invading pathogens and set the stage for tissue recovery, regeneration, and remodelling, once the instigating factors are removed from the body (Attiq et al., 2018). However, unresolved inflammation that arises from persistent inflammatory stimuli and perturbation in regulatory mechanisms have pathological consequences (including organ failure, fibrosis, autoimmunity and metaplasia), which increases the risk of cancers in selected organs (Medzhitov, 2008).

Over the past few decades, evidence gathered from a range of molecular studies using genetically modified mice to epidemiological data of patients has supported the functional relationship between inflammation and cancer, which also led to general acceptance of tumour-promoting inflammation as a key hallmark of cancer (Hanahan and Weinberg, 2011; Yu et al., 2018). Of interest, hallmarks of tumour-promoting and cancer-associated inflammation are striking similar, sharing a complex network of stromal (fibroblasts and vascular cells) and inflammatory immune cells that collectively form the tumour microenvironment (TME). Inflammation momentously orchestrates the TME composition and cellular plasticity in chronic inflammatory and tissue repair processes, also when observed in the context of cancer-induced smouldering inflammation. Regardless of cancer-instigating factors, innate immune cells and inflammatory cytokines made up the composition of TME, suggesting that the innate immune system plays a vital role at every step of carcinogenesis (Mantovani et al., 2008; Greten and Grivennikov, 2019).

As part of the evolutionary development, cross-talk interactions between signalling pathways were developed to capacitate the cells to perform homeostasis without glitches. Nonetheless, the mechanistic studies focusing on the pro-tumorigenic inflammatory pathways have suggested that tumour cells have shown the ability to usurp the homeostasis-promoting pathways for their benefit (Greten and Grivennikov, 2019). In this scenario, feedback and cross-talk mechanisms previously dubbed as an evolutionary gift become liabilities instead. It became evident in cancer treatment that the inhibition of one cancer-associated signalling pathway can give rise to a secondary survival pathway that potentially encumbers cancer drug efficacy and propagates chemotherapeutic resistance (Prahallad and Bernards, 2016). Hence, transcriptional, and regulatory signalling mechanisms propagating the plasticity of the stromal, tumour, and inflammatory immune cells in TME need to be elucidated for a better understanding of complex cross-talk interactions and functional diversity of multiple cell types in tumour-associated inflammation. Here, we review the molecular pathways that establish the connection of inflammation with cancer and describe their role in abrogating antitumour immunity during tumour development and progression. We also discussed strategies focusing on the cross-talk interaction of signalling pathways to harness synthetic lethal drug combinations for cancer treatment.

## 2 Tumour-promoting inflammation; extrinsic and intrinsic pathways

While few studies have casually implicated inflammation for urinary bladder, gastric, hepatic and colon cancer, others formally substantiated its role in the development of prostate, pulmonary, pancreas and oesophageal cancer. It is worth mentioning that in most cancers, environmental variants, including food, pollutants, and infections agents, serve as instigating factors. Down the lane, inflammation corroborates with ecological exposures and bolsters their cancer potential (Groopman and Kensler, 2005). The underlying molecular mechanisms of inflammation-induced cancers are intricate and involve both innate and adaptive immunity (Condeelis and Pollard, 2006; De Visser et al., 2006). Even though viral oncogene directly participates in the neoplastic transformation, no evidence supports that infection or pathogen-encoded oncogenes are the prerequisites for inflammatory cells to induce cancer (Chisari, 2000). In epithelial cells, the release of highly reactive oxygen and nitrogen species by phagocytic inflammatory cells poses direct oxidative or nitrosative damage to the DNA. In contrast, free-radical chain reactions with the phospholipid and other cellular components cause additional indirect damage (Ames et al., 1995). Consequently, the necrosed cells are substituted with resident progenitor and stem cells to conserve the functional barrier of epithelial cells. In this scenario, the risk of mutation significantly increases in the epithelial cells, especially those that undergo DNA synthesis in the presence of the said damaging factors. Here, the cytokines, including IL-1β, IL-8, IL-10, VEGF and TGF-β released by the inflammatory cells promulgate proliferation and induce angiogenesis, initiating the neoplastic transformation of the epithelial cells (Coussens et al., 2000).

Proteolytic enzymes produced by the inflammatory cell are pivotal for the disease progression. It allows them to migrate through the extracellular matrix and promotes the invasion of epithelial cells into stromal and vasculature compartments for tumour metastasis (Condeelis and Pollard, 2006). According to Dranoff (2004), aberrated cytokine levels, which includes insufficient production of anti-inflammatory and excessive secretion of pro-inflammatory cytokines, promote inflammation and cancer. Moreover, suppression of immune-surveillance modulating cell-mediated anti-tumour activities allows the cancer cell to evade the immune responses and proliferate without any check and balance (Langowski et al., 2006).

**FIGURE 1:**
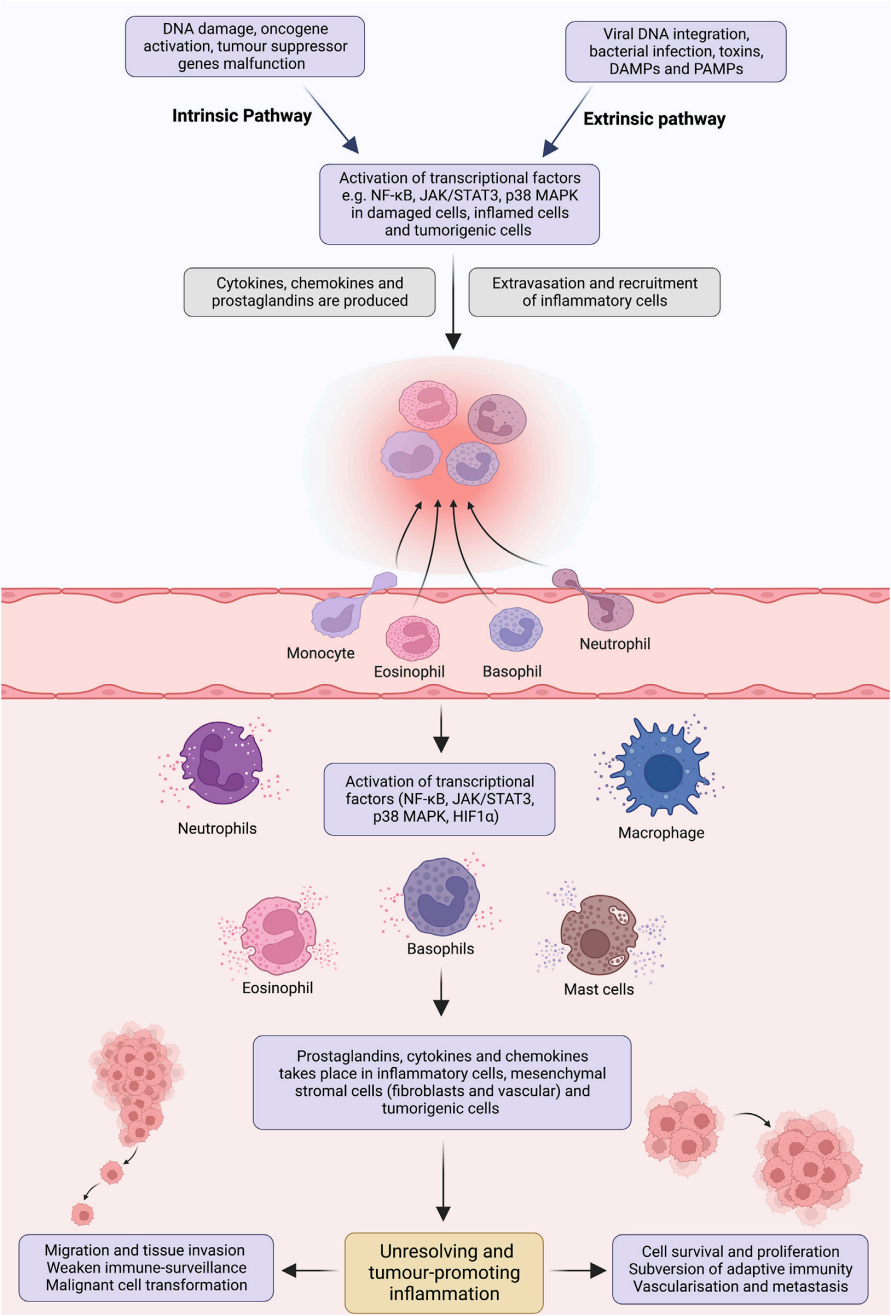
Pathways involved in tumour-promoting inflammation. Insufficient mechanisms to resolve inflammation and perseverance in instigating stimuli result in non-resolving inflammation. Development of angiogenesis to support the tissue remodelling, infiltration of tumour-associated macrophages, immature myeloid and T cells, abundance of inflammatory mediators and imbalance of pro- and anti-inflammatory cytokines creates an ideal microenvironment for non-resolving inflammation. Extrinsic and intrinsic pathways bridge inflammation and cancer. In the extrinsic pathway, an inflammatory response is triggered by exogenous factors, including the PAMPs from pathogens or DAMPs from necrotic cells, which activate the inflammatory cells and establish a microenvironment that potentiates cancer risk. On the contrary, the intrinsic pathway is induced by a mutation in cancer-associated genetic factors such as activating proto-oncogenes, tumour suppressor and DNA repair genes inactivation and chromosomal aberration. These events pave the way for inflammatory microenvironment and neoplastic transformation by upregulating the expression of inflammation-related programs. These two pathways congregate and activate the transcriptional factors (e.g., NF-κB, STAT3, HIFα), which propagate the production of inflammatory mediators, including cytokine, chemokines, COX2/PGE_2_ and ROS, in inflammatory, stromal, and tumour cells. Perpetually activated transcriptional factors orchestrate pro-tumorigenic inflammatory microenvironment, which is well-known for its tumour-promoting effects.

Infiltration of the immune cells (e.g., tumour-associated macrophages, immature myeloid cells, T cells), tissue remodelling, imbalance of inflammatory mediators and anti-inflammatory mechanisms altogether make up tumour-promoting inflammation (Yu et al., 2018). The development process can be curtailed into two pathways; extrinsic and intrinsic. In the extrinsic pathway, exogenous factors, including pathogen-associated molecular patterns (PAMPs) and damage-associated molecular patterns (DAMPs), initiate inflammatory responses via activating inflammatory cells, increasing the risk of cancer (Mantovani et al., 2008; Takeuchi and Akira, 2010). Nevertheless, the intrinsic pathway is mediated by genetic alterations, affecting the functionality of oncogenes, including proto-oncogenes, tumour suppressor genes and DNA repair genes (Vogelstein and Kinzler, 2004). This orchestrates the development of microenvironment and inflammation-related programs, which serves as prerequisites for neoplastic transformation (Figure 1).

## 3 Key orchestrators of tumour-promoting inflammation

**FIGURE 2:**
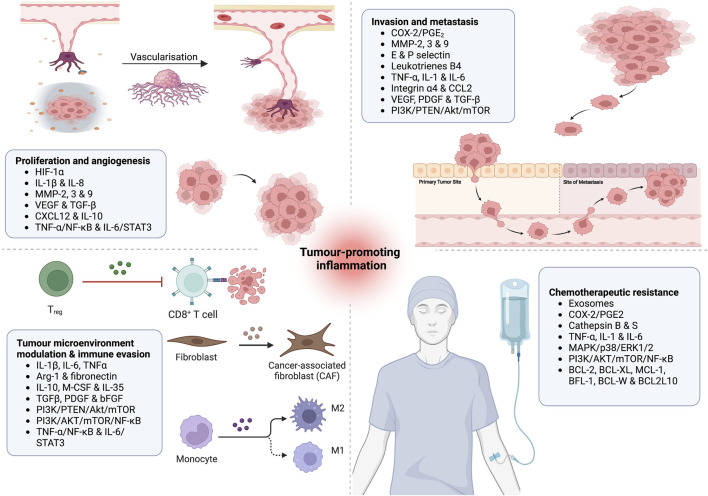
Putative role of inflammatory effector molecules in (1) tumour proliferation and angiogenesis; (2) tumour metastasis and invasion; (3) modulation of tumour microenvironment and immune evasion; and (4) induction of chemotherapeutic resistance.

Macrophages have a diverse phenotypic spectrum allowing them to participate and play an essential role in various inflammatory signalling pathways. They are responsible for phagocytosis, antigen presentation and immunomodulation (Arango Duque and Descoteaux, 2014). Varying sizes and compositions of leukocytes infiltrate, containing tumour-associated macrophages (TAM), mast, hematopoietic, and T cells have been detected in most tumours. TAMs play the most significant role in tumour growth by suppressing innate immunity and promoting tissue remodelling (Qian and Pollard, 2010; Yahaya et al., 2019). TAMs secrete proangiogenic mediators such as vascular endothelial growth factors (VEGFs), promoting angiogenesis required by tumours of specific size. They can also inhibit the anticancer immune responses by releasing immunosuppressive factors such as IL-10, transforming growth factor (TGF) and prostaglandin E_2_. Additionally, by activating arginase 1 (ARG1), TAMs decrease the availability of L-arginine and promote proapoptotic activity in the T cells (Ostuni et al., 2015). Poor prognosis and a reduced survival rate have been observed among classic Hodgkin’s lymphoma patients with elevated TAM levels, suggesting its active role in the disease’s development and progression (Figure 2) (Steidl et al., 2010).

Cytokine is a big family of small proteins involved in intracellular communication, immunomodulation and cellular plasticity. Cytokines can be broadly classified into interleukins (IL), chemokines (CK), tumour necrosis factor (TNF), interferons (IFN) and tumour growth factor. Upon receiving the stimulus, peripheral cells instantaneously produce and release these biogenic proteins (Dembic, 2015). IL-1 is one of the most potent immunomodulator, promoting inflammation, thermoregulation, wound healing and haemostasis (Attiq et al., 2021b). Moreover, it facilitates the infiltration of phagocytes and fibroblasts into the malignant cells, promoting mutagenesis, ROS production and tumour growth (Dembic, 2015). IL-6 increases the production of acute-phase proteins and propagates specific cellular and humoral immune responses by activating T cells and recruiting monocytes at the inflammation site (Gabay, 2006; Attiq et al., 2021a). Additionally, it promotes cell proliferation, differentiation, angiogenesis and antiapoptotic activities by inducing VEGF synthesis (Setrerrahmane and Xu, 2017). Upregulated IL-1 and IL-6 expressions are frequently observed in tumours like breast cancer, highlighting their anti-apoptotic and pro-survival properties at all stages of cancer (Garcia-Tuñón et al., 2005).

TNF serves as a master regulator of inflammatory mediators (Parameswaran and Patial, 2010). Based on its pleiotropic nature, it is classified into TNF-α and TNF-β. TNF receptor 1 (TNFR) activation by TNF-α increases the turnover of transcriptional factors, including NF-κB, and triggers inflammatory cascades. In comparison, TNFR-2 activation induces endothelial adhesion, vascular permeability and tissue regeneration (Yang et al., 2018). Numerous studies have implicated TNF-α for tumour progression based on its potential to encourage the mass migration of myeloid cells into the microenvironment and promote vascularisation via increasing the production of VEGF. Moreover, the activation of transcription factors (NF-κB, AP-1 and ELK-1) allows TNF-α to actively participate in malignant cell proliferation (Waters et al., 2013). The late study of Ferrajoli et al. (2002) used TNF-α as a tumorigenesis biomarker and associated its elevated plasma level with varying stages of tumour progression and metastasis in chronic lymphocytic leukaemia.

The chemokines family is functionally divided into inflammatory and homeostatic chemokines. The former recruits the leukocytes towards injury and inflammation, while the latter directs the leukocytes to support haematopoiesis (Chen et al., 2018). Nevertheless, the recruitment of effector T cells in autoimmune diseases is also the consequence of continuous chemokines production (Moser and Willimann, 2004). Chemokines contribute to tumour growth by activating mitogen-activated protein kinase (MAPK), responsible for upregulating the expression of growth-stimulating genes. In terms of vascularisation, CXCL12 is the most potent angiogenic chemokine, which enables the adequate amount of oxygen supply required in primary and metastatic breast cancer (Chow and Luster, 2014).

The growth factors are produced by mononuclear cells to recruit, stimulate and proliferate the fibroblasts and endothelial cells. Inflammatory cell-derived peptide growth factors are platelets-derived growth factor (PDGF), transforming growth factor (TGF) β, epidermal growth factor (EGF) and vascular endothelial growth factor (VEGF). PDGF is involved in stimulating vascular smooth muscle cell migration and proliferation (Figure 2). TGF-β promotes chemotaxis, leukocyte recruitment and adhesion. Dermal regeneration is the primary function of the EGF; nevertheless, it also supports proliferation and migration of keratinocytes, endothelial and fibroblast cells (Bodnar, 2013; Mukai et al., 2018). VEGF increases vascularity, activates T cells and promotes endothelial cell proliferation and migration (Angelo and Kurzrock, 2007). Due to their definitive role in proliferation, survival and micro-metastasis, it is unsurprising that these growth factors have directly associated with cancer progression. For instance, upregulated expressions of EGF and VEGF and their receptors are extensively reported in colorectal carcinoma (Sasaki et al., 2013). Although TGF-β and PDGF are generally known for growth-suppressing activities, studies have suggested that successive genetic mutations and DNA damage alter their signalling hierarchy to promote malignant transformation and survival in colorectal and breast cancers (Farooqi and Siddik, 2015).

## 4 Signalling pathways; tumour and inflammation rendezvous

### 4.1 Cyclooxygenase pathway

The cyclooxygenase enzymes convert arachidonic acid into prostaglandins (PGs) and stimulate the production of inflammatory chemokines and cytokines. The mammalian cyclooxygenases are classified into COX-1 and COX-2 (Attiq et al., 2017). The presence of a 3′-untranslated instability sequence makes the COX-2 mRNA turnover relatively quicker than COX-1. For the same reason, COX-2 is inducible and participates in the development of inflammatory disorders and tumour-promoting inflammation (Attiq et al., 2018; Attiq et al., 2021a). Upregulated COX-2 expressions are frequently reported in epithelial and stromal tumours, including prostate, colorectal and non-small cell lung cancer (NSCLC) (Sandler and Dubinett, 2004; Wu and Sun, 2015). These expressions harness the hypoxic condition causing the dysregulation of the yes-associated protein 1 (YAP-1), which regulates the transcription of genes involved in cell proliferation and apoptotic genes suppression (Hashemi Goradel et al., 2019).

Immunosuppressive properties of COX-2 are driven by its major metabolic by-product, PGE_2_. Cytosolic PGE_2_ synthase (cPGES) and membrane-bound microsomal PGE_2_ synthase-1 and -2 (mPGEs-1 and -2) participate in the catalytic conversion of PGH_2_ to PGE_2_. Once synthesised, PGE_2_ binds and activates respective membrane receptors (EP1-4) (Reader et al., 2011). The coupling of EP-1 with the Gq-phospholipase C (PLC)-inositol trisphosphate (IP3) pathway increases the intracellular concentration of Ca^2+^. EP2 and EP4 receptors are coupled with the Gs-adenylyl cyclase (AC)-cAMP-protein kinase A (PKA) pathway. Pertussis toxin-sensitive Gi protein-bound EP3 decreases the cAMP and inhibits of concentration of AC (Liu et al., 2015). Immunohistochemistry of colorectal cancer cells exhibits 100-fold of EP4 than the normal colonic epithelium, indicating an association between PGE_2_ signalling and colorectal tumorigenesis (Fujino et al., 2002). Furthermore, cAMP/protein kinase A (PKA) dependent mechanism is the main activating factor of EP2 receptor-mediated Tcf transcriptional activity. On the contrary, EP4 receptor-mediated activation occurs primarily through a phosphatidylinositol 3-kinase (PI3K) dependent pathway (Fujino et al., 2003). Another study has reported the activation of a PI3K-dependent pathway by PGE_2_, which resulted in the signal-regulated kinases (ERKs) phosphorylation and subsequent induction of the functional expression of early growth response factor 1 (EGR-1). Interestingly, this activation cascade was only observed in cells expressing EP4 and was completely absent in cells with EP2 (Guillemot et al., 2001). It is worth mentioning that EGR-1-derived PI3K- and ERK-dependent pathway controls the key regulator of cyclinD1. Moreover, EP4-derived PI3K/Akt activation stimulates the proliferation and motility of colorectal cancer cells, suggesting a potential role for EP4 in development and progression of cancer (Sheng et al., 2001; Sonoshita et al., 2001).

COX-2 and PGE_2_ weaken immune surveillance by evading the activated macrophages and cytotoxic T cells, allowing maximal tumour cell expansion without check and balance (C. S. Williams, 1999). Moreover, by activating the EP2 and EP4 receptors of natural killer cells (NK), tumour-derived PGE_2_ inhibit NK cells to migrate, exhibit cytotoxic effect and producing interferon-gamma. Frondoside A, an EP4 inhibitor, suppresses breast cancer metastasis by abrogating PGE_2_ production and augmenting NF-dependent IFN-γ synthesis (Gualde and Harizi, 2004; Martinet et al., 2010). In the same manner, COX-2/PGE_2_ nexus also suppress the maturation of dendritic cells and their major histocompatibility complex (MHC) expression, significantly abrogating their function to present antigen and activating T cells (Harizi et al., 2003). These suppressive behaviour are driven by PGE_2_-induced IL-10 production and EP2 and EP4 DC receptor activation. Hence, studies have suggested that targeted therapies focusing on the EP2/EP4 signalling can be attractive therapeutic interventions to reactivate DC immune activities (Harris et al., 2007). Cross-talk of numerous signals is responsible for regulating the functionality of COX-2 on the cancer cells. According to Hashemi Goradel et al. (2019), nuclear factor-kappa β (NF-κB), EGFR and mitogen-activated protein kinase (MAPK) transcriptional proteins are reported to upstream COX-2 expression in various cancers (Hashemi Goradel et al., 2019).

### 4.2 Nuclear factor kappa-light-chain enhancer of activated B Cells (NF-κB)

The NF-κB transcriptional family consist of RelA (p65), RelB, and c-Rel. NF-κB1 (p105) and NF-κB2 (p100) precursor proteins are processed to p50 and p52, making up the Rel homology domains responsible for binding and dimerization of DNA. Inhibitory IκB serves as negative regulators under resting conditions, restricting the NF-κB dimers to the cytosol (Attiq et al., 2021b; Attiq et al., 2021c). Once the stimulus is received, IκB kinase (IKK) complex undergoes phosphorylation, proteasomal degradation and ubiquitination. This results in the release of bound NF-κB dimers and paves the way for nuclear translocation. The binding of NF-κB to the DNA activates its transcriptional activity, increasing the production of cytokines, chemokines and growth factors involved in cellular communication, differentiation, proliferation, survival, and immunomodulation (Yao et al., 2019; Jalil et al., 2020; Attiq et al., 2021c). Hence, it is unsurprising that NF-κB dysfunction is implicated in inflammatory disorders, autoimmune diseases and tumorigenesis.

The late discovery of homologue cRel encoded retroviral oncogene v-Rel, established NF-κB role in oncogenesis (Gilmore, 2004). The mutated genes of NF-κB subunits or IκB proteins are frequently observed in numerous malignant cells. Mutated and fused genes of IKKA responsible for the activation of IKKα are frequently observed in breast cancers. These aberrations promote the self-renewal of cancer progenitors and augment pro-carcinogenic effects of progesterone in breast cancer (Stratton et al., 2009). Evidence supports that constant activation of NF-κB is more prevalent in tumours than subfraction of malignancies with confirmed mutations in NF-κB or IκB-encoding (Ben-Neriah and Karin, 2011). Hyperactivation of NF-κB signalling has the potential to promote atypical cell proliferation, differentiation, metastasis and chemotherapy resistance in breast, colon and lymphatic cancer (Walther et al., 2015; Forlani et al., 2016; Sau et al., 2016). Similar activation is observed in Epstein–Barr virus (EBV)-induced T- and NK-cell neoplasms. Interestingly, LMP1 viral protein closely resembles the TNF-receptors domains. It interacts with TRAF and TRADD signal transducers (Luftig et al., 2004; Kieser, 2008) and promotes EBV-positive T- and NK-cell neoplasms via NF-κB activation (Takada et al., 2017). Greten et al. (2004) suggested that conditional silencing of intestinal epithelial IKKβ is effective in suppressing the NF-κB activation and disease progression in colitis-associated colon cancer.

Silencing of BRCA1-induced phosphorylation of Ser536 site of p65 and p100/p52 activates the NF-κB canonical (p65/p50) and non-canonical pathway (p100/p52), nuclear translocation, p52/RelB coupling and proliferation of MCF1 breast cancer cell lines (Sau et al., 2016). Mesenchymal trans-differentiation and radiotherapy resistance increase with MLK4 binding and IKKα phosphorylation and NF-κB nuclear translocation in glioma stem cells (Kim et al., 2016). LMP1-induced mTORC1 activation plays a significant role in modulating NF-κB pathway in nasopharyngeal carcinoma cells (NPC). Moreover, Glut1 transcription inhibition and LMP1-induced inactivation negatively affect the aerobic glycolysis in MTORC1 gene knockout NPC HONE1, which also defines the NF-κB role in regulating energy metabolism required for the cancerous survival and growth, as observed in NPC (Zhang et al., 2017).

**FIGURE 3:**
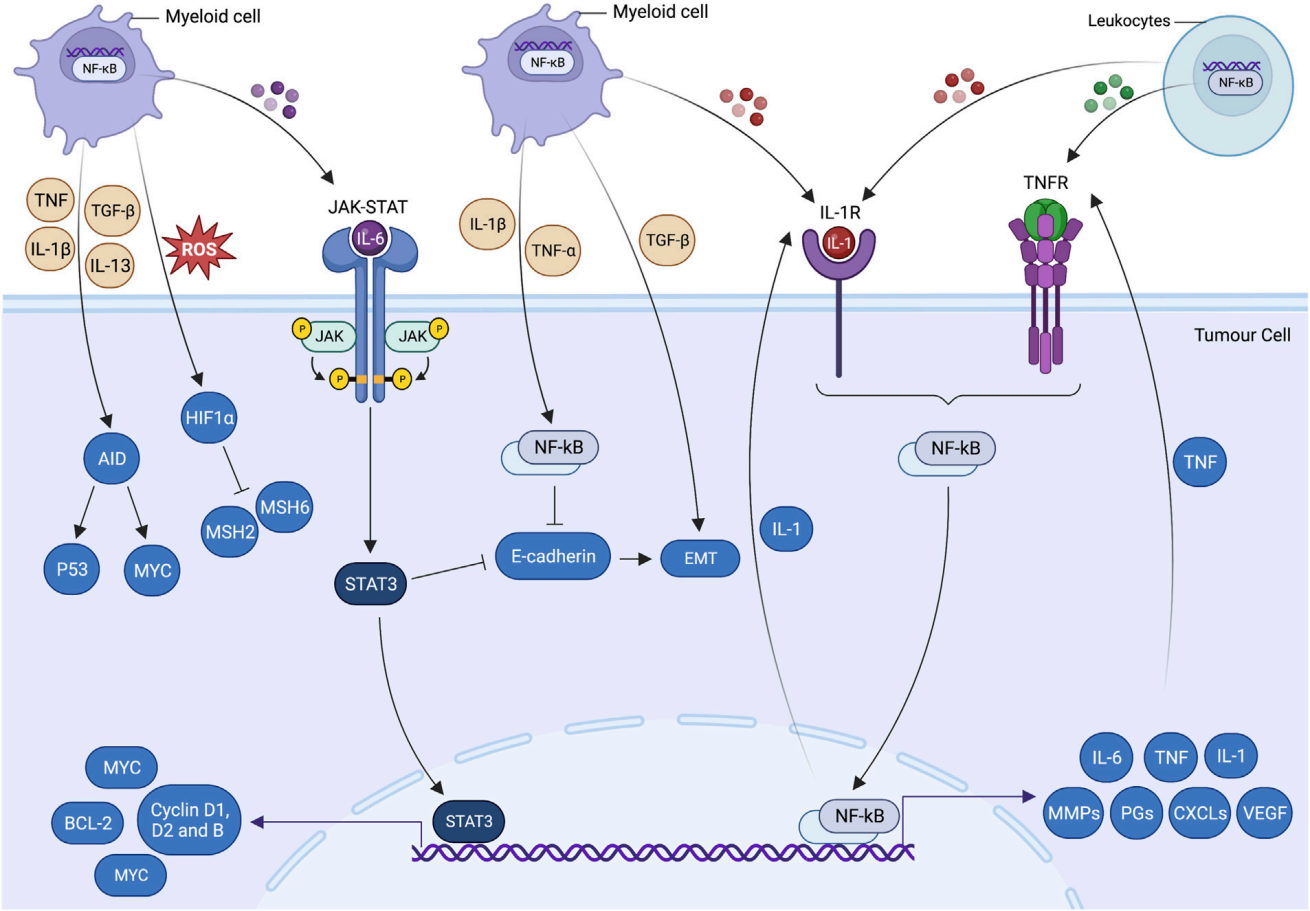
Inflammatory cell signalling pathways and tumorigenesis. Cross talk of cell-centric and -extrinsic interactions pave the way for tumorigenesis. These processes propagate cancer-associated mutations, which give rise to genetic volatility, hyperproliferation, restructured stromal milieu and poorly differentiated states of epithelial and mesenchymal cells. Inflammatory cells have a unique potential to trigger essential molecular processes that are prerequisites of tumorigenesis. Inflammatory cytokine produced by tumour-infiltrating myeloid cells allows cancer cells to evade apoptosis and promote cell growth via activating transcriptional factors, including signal transducer and activator of transcription 3 (STAT3) and nuclear factor-kappa B (NF-κB). Activated STAT3 and NF-κB coordinate the production of IL-6 and transforming growth factor beta (TGF-β), which downregulates the epithelial differentiation markers expression (e.g., E-cadherin) and promotes epithelial-mesenchymal transition (EMT). Genetic instability is augmented by hypoxia-induced suppression of DNA repair mechanisms and ectopic expression of activation-induced cytidine deaminase (AID). IL-6-induced STAT3 activation by myeloid cells supplements the pre-malignant cell proliferation and anti-apoptotic activities by upregulating the expressions of the proto-oncogene MYC and cell cycle regulators such as cyclin D1, cyclin D2 and cyclin B. MYC-induced BCL2 and BCL2-like1expressions are also responsible for significantly increasing the cell survival and anti-apoptotic activities. Likewise, IL-1α/IL-1R (TNF receptor) autocrine loop signalling and myeloid differentiation primary response 88 (MYD88) results in the activation of NF-κB. MYD88 also actively participate in the development and expansion of tumours by controlling the production of IL-6 in leukocytes. The tumour-promoting role of NF-κB (cell-centric and -extrinsic) during inflammation is further validated by the Toll-like receptor 2 (TLR2)-mediated STAT-3 activation in gastric tumorigenesis.

Hypoxia-inducible factor 1-alpha (HIF-1α) and NF-κB mediators, including cytokines, matrix metalloproteinase (MMP) and oncogenes, increase the VEGF production and support tumour angiogenesis (Forsythe et al., 1996). NF-κB-driven MMP-2, MMP-3 and MMP-9 potentially degrade the basement membrane, perform remodelling of the extracellular matrix and facilitate angiogenesis and metastasis of endothelial cells and tumour cells, respectively (John and Tuszynski, 2001), On the contrary, NF-κB inactivation is reported to abrogate the production of VEGF, supplementary fibroblast growth factor (bFGF), IL-8 and MMP-9 (Huang et al., 2001). For tumour metastasis, subsequent activation of NF-κB, TNF-α and Twist1 is required to modulate the epithelial-mesenchymal transition (EMT) (Figure 3). COP9 signalosome 2 (CSN2) is known for its role in ubiquitin-proteasome pathway stabilises snail (zinc-finger transcription repressor) and activates NF-κB in inflammation-induced cell migration and invasion (Wu et al., 2009). Hiratsuka et al. (2006) further suggested that serum amyloid A3 (SAA3)-TLR4 signalling-induced NF-κB activation establishes an inflammatory state, which facilitates the metastasis of epithelial and myeloid cells in premetastatic lungs. Total suppression of NF-κB via upregulation of IκBα super-repressor or silencing RelA/IKK2 has shown promising results in reducing the tumour size. Nevertheless, no alternation in apoptotic pathways was observed in KRAS-induced lung adenocarcinoma (Bassères et al., 2010). Moreover, deletion of IKK2 and NF-κB inhibition exhibit impaired tumour cell proliferation and KRAS-ERK-NF-κB-Timp1-CD63-FAK-ERK positive feedback loop in pulmonary cancer animal model (Xia et al., 2012).

### 4.3 Janus Kinase (JAK)/signal transducer and activator of transcription (STAT) pathway

The JAK family consists of JAK1, JAK2, JAK3 and TYK2. Although the two tyrosine kinase domains JH1 and JH2 are laid adjacent to each other but in terms of functionality, they are discrete. JH1 carry out the phosphorylation and activate the pathway, whereas JH2 reciprocally regulate the functions of JH1. Inactive JAK dimers are part of the cell surface complexes, which closely bind to transmembrane region of the receptors. Tyrosine residues present at the intracellular receptor tail are phosphorylated by activated JAKs. The binding of legend to the receptor alters the JAKs positioning, brings the confirmational change and results in phosphorylation and activation of the tyrosine kinases (Brooks et al., 2014). Transcriptional protein family of STAT consist of STAT1, STAT2, STAT3, STAT4, STAT5A, STAT5B and STAT6. Cytosolic STATs serve as a JAK substrate and avidly bind with the phosphorylated receptors (Kaplan, 2013). Phosphorylated STAT form dimers and translocate into the nucleus and upregulate the expression of transcriptional genes (Thomas et al., 2015). JAK/STAT pathway regulates numerous physiological functions such as embryonic development, stem cell modulation, haematopoiesis, inflammation, cell survival, proliferation, differentiation, and apoptosis (Richard and Stephens, 2014; Garrido-Trigo and Salas, 2020).

Activated JAK/STAT has been detected frequently in cancer-related inflammation; however, the underpinning for dysregulated activation has yet to be identified. IL-6 serves as a key stimulator of STAT3. The binding of IL-6 allows phosphorylated STAT3 to completely activate the NF-κB (Figure 3) (Jin et al., 2013). Activated transcription factors translocate into the nucleus and promote the production of cytokines, chemokines and growth factors involved in tumour proliferation, survival and angiogenesis (Fan et al., 2013). Accordingly to Yu et al. (2009), STAT5 and STAT6 have been associated with to upregulation of the genes required for haematopoietic tumour survival and proliferation. STAT4 increases cancer-associated fibroblasts (CAF) that supports the production of CXCL12, IL-6 and VEGF and promotes the metastasis of ovarian cancer (Zhao et al., 2017). Although, STAT1 and STAT2 are known for fostering smouldering inflammation in TME by increasing the production of inflammatory cytokines and chemokine (Zhang et al., 2017). Contrarily, Stephanou and Latchman (2005) suggest that the accumulation of STAT1 and STAT2 in the nucleus encourages cell cycle arrest and bolsters anti-tumour innate immunity.

In atopic dermatitis model, JAK/STAT is reported to increase the VEGF production from mast cells and promote angiogenesis (Bao, Zhang and Chan, 2013). The oncogenic effect of JAK starts with the gain-of-function mutations that result in the activation of pathways involved in haematological malignancies. These mutations augment the phosphorylation of JAK1 and STAT3 and promote cytokine-dependent growth. Additionally, STAT3 prevents apoptosis by activating cyclin-dependent kinases (CDKs), which increase the transcription of positive regulators such as cyclin D2 and downregulate transcription of CDK inhibitors such as p21 (Figure 3). Together with STAT5, they activate transcription of Bcl-x, producing the anti-apoptotic protein Bcl-x_L._ As the Bcl-x_L_ production goes up, it facilitates the tumour cells to adapt to a hypoxic environment, promotes angiogenesis and suppresses anti-tumour immune responses (Thomas et al., 2015). It is worth mentioning that JAK2, an autoregulator of JAK/STAT pathway, has been excessively reported in the chromosomal locus of gastric adenocarcinoma. As the JAK2 activity increases, it reciprocates the JAK/STAT turnover and simultaneously induces phosphorylation of STAT5, which is an important mediator for the micro-metastasis, tumour invasion and expansion (Bass et al., 2014; Pencik et al., 2016).

### 4.4 Mitogen-activated protein kinases (MAPKs)

MAPKs are a family of highly conserved serine or protein kinases involved in various processes such as gene induction, cell proliferation, differentiation, cellular stress and inflammatory responses (Zhang and Liu, 2002; Kostenko et al., 2011). MAPKs are classified into four major kinases groups; the extracellular signal-regulated kinases (ERKs), also known as p42/44 MAP kinase, c-jun N-terminal kinase (JNK), commonly referred to as stress-activated protein kinase-1 [SAPK1] and Big MAP kinase (BMK) alternatively known as ERK5 and p38 MAPK (Koul et al., 2013). Mitogens serve as the activator of primary transducers ERK1 and ERK2, which promote cell proliferation and growth upon activation. Oxidative stress and growth factors serve as the primary inducer of BMK. JNK/SAPKs and p38 respond profoundly to stress signals and inflammatory cytokines, but unlike other kinases, they respond poorly towards growth factors (Vassalli et al., 2012).

The signal transfer between MAPK and upstream kinase (MAPKK) is highly specific in nature. MAP/ERK kinase (MEK) 1 and 2 phosphorylates p42/p44 (ERK) MAP kinases. MAP kinase kinase (MKK) 3 and 6 exclusively activate p38 MAP kinase. Whereas MKK7 and MKK4 only activate JNK. However, in exceptional cases, MKK4 gains add-on potential to activate p38 MAP kinase based on its overexpression (Vander Griend et al., 2005). 4p38 MAPK family members are phosphorylated by MKK6, where MKK3 phosphorylates all p38α, p38γ, and p38δ expect p38β. These stimuli are received at the plasma membrane, which results in the activation of MAP kinase kinase kinase (MKKK). MKKK further activates the MKK, which consequently ends up activating MAPK (Cuadrado and Nebreda, 2010). During this process, the threonine and tyrosine residues in a TXY motif of the MAPK are dually phosphorylated by the MKK. Activated MAPK targets and process MAPKAP-K2, c-Jun and c-myc, c-Jun, Tau and IκBα for p38, ERK and JNK, respectively (Boutros et al., 2008). Whereas the mitogen-activated protein kinase phosphatases (MKPs) serve as the negative regulator that suppresses the kinases activity under resting conditions. Of interest, altered expression of MKPs, including MKP-1, MKP-2 and MKP-3, have been frequently reported in non-small cell lung cancer and breast cancers (Wang et al., 2019), where other studies have associated these aberrations with chronic inflammatory disorders, autoimmune diseases, and tumour-promoting inflammation (Wu, 2007; Wancket et al., 2012; Lang and Raffi, 2019).

According to Thalhamer et al. (2008), proinflammatory mediators, including TNF-α, IL-1 and IL-6 serve as major inducers of MAPK pathway. It is also suggested that ERK-MAPK regulates IL-6, IL-12, IL-23, TNF-α biosynthesis and promotes phospholipase A2 induction, PGs production and chemotaxis of immune cells, creating an inflammatory microenvironment for tumorigenesis. JNK-MAPK regulate the expressions and activation of TNF-α, IL-2, E-selectin and MMPs. MAPKs, p38 serves as the master activator and recruiter of leucocyte and regulates the expressions of IL-6, IL-8 and COX-2 (Kaminska, 2005; Huang P. et al., 2010). The ERK signalling pathway promotes cancer cell migration through the phosphorylation of myosin and light chain focal adhesion kinases. JNK-MAPK pathway ensures cancer cell survival by increasing the anti-apoptotic protein MCL-1 production. Besides that, MMPs induced by JNK help in the degradation of extracellular matrix proteins, which facilitate tumour invasion and expansion through metastasis (Kim and Choi, 2010). On the contrary, p38 exhibits a dual function as it can suppress and simultaneously promote cancer development (Koul et al., 2013). Its activation leads to the epithelial-mesenchymal transition (EMT) of cells in the primary tumour, which enhances their invasion potential and makes extravasation easier for the migrating tumour cells (Bhowmick et al., 2001). Cheng et al. (2004) suggested that inhibition of p38 MAPK augment the circulating cancer cell’s survival by enhancing their resistance to anoikis (Cheng et al., 2004). The low to high activity ratio of p38 and ERK1/2 pathway is one of the significant contributing factors for tumour cell dormancy. In contrast, tumour suppressor activity of p38 MAPK has also been highlighted by studies which analysed the phenotype of mice with disrupted MEK3, MEK6 and p38α genes. Interestingly, the fibroblast cells of these animal models exhibited enhanced transforming potential of oncogenes. Likewise, the tumorigenic potential in nude mice was also recorded exceptionally high (Bulavin et al., 2002; Brancho et al., 2003; Bulavin and Fornace, 2004; Timofeev et al., 2005). Down the lane, the p38 tumour-suppressing function upregulated p53 activation, potentiates p53-induced apoptosis, and serves as a cell cycle negative regulator (Bradham and McClay, 2006).

### 4.5 PI3K/Akt/mTOR pathway

Phosphatidyl-inositol-3-kinases (PI3Ks) are kinases with inositol 3′-OH moiety attached to inositol phospholipids. PI3K catalytic and regulatory subunits are known as class I PI3Ks heterodimers that are further subdivided into IA (PI3Kα, β, and δ) and IB (PI3Kγ). Former is activated by the tyrosine kinase coupled receptors, while later is activated by G proteins coupled receptors (Akinleye et al., 2013). Autophosphorylation of the tyrosine residues results in the activation of tyrosine kinases of growth factor receptors. PI3K binds with phosphotyrosine consensus residues. Interaction of SH2 domains with the growth factor receptor adaptor subunit results in CAT subunit allosteric activation and PI3K enrolment to the plasma membrane. The phosphatidylinositol-4,5-bisphosphate (PI-4,5-P_2_) responds to PI3K and activates the second messenger phosphatidylinositol-3,4,5-triphosphate (PI3,4,5-P_3_) (Waugh et al., 2009). PI3,4,5-P_3_ synthesis increases serine/threonine kinase-3′-phosphoinositide-dependent kinase 1 (PDK1) and Akt/protein kinase B (PKB) containing pleckstrin homology (PH) domains. Together, Akt/PKB regulates the cell cycle, survival and proliferation. Additionally, activated Akt/PKB is also implicated for maintaining the inflammatory cells activity within the TME (Vara et al., 2004; Tang et al., 2018).

Akt kinases are functionally related to AMP/GMP and protein kinase C which falls under the umbrella of the AGC kinase family. Akt kinases consist of three common domains, including the N-terminal PH domain, central kinase CAT domain and C-terminal extension (EXT) containing a regulatory hydrophobic motif (HM) (Kumar and Madison, 2005). Activated Akt/PKB significantly hampers the activities of pro-apoptotic factors, including Bad and Procaspase-9, promoting anti-apoptotic activity and cell survival. In addition, it abrogates TNF-induced apoptosis, which consequently increases the apoptotic resistance of prostate cancer cells (Chen et al., 2001). The parallel activation of IκB kinase with Akt/PKB creates a cross-talk between NF-κB and PI3K/Akt/mTOR pathways, which has been reported to aggrandize the inflammogenesis and anti-apoptotic activity in lymphoma cells (Hussain et al., 2012; Li et al., 2018). With regards to cell cycle progression and growth, Akt modulates protein synthesis and glycogen metabolism via interacting with glycogen synthase kinase-3 (GSK3) and mammalian target of rapamycin (mTOR). Regulation of G1/S cell cycle progression is positively controlled by Akt/PKB cascade via inactivating GSK3-β. This inactivation results in the complete abrogation of fork head family transcription factors and tumour suppressor tuberin (TSC2). Increased cyclin D1 production alleviates p27Kip1, promoting cell cycle and growth progression (Liang and Slingerland, 2003). Interestingly, PI3K/AKT is crucial for macrophage survival, migration and proliferation in cancer-related inflammation (Vergadi et al., 2017). Likewise, PI3K is a key player in regulating the extravasation and migration of innate immune cells to the inflammatory microenvironment of various tumours (Hawkins and Stephens, 2015). The gene amplification of the PI3K and AKT is said to increase the incidences of cervix and ovarian cancers, while the amplification of the AKT2 gene is commonly observed in ovarian, pancreas, breast and gastric tumours (Vara et al., 2004).

mTORC1 and mTORC2 complexes modulate the activity of the mTOR pathway. Raptor, mLST8 and PRAS40 make the mTORC1 complex highly sensitive to rapamycin and first-generation mTOR inhibitors (Szwed et al., 2021). On the contrary, mTORC2 consists of mTOR, Rictor, Sin1, and mLST8. Due to a change in configuration, mTORC2 losses its rapamycin sensitivity but still retains its contribution to numerous cellular processes (Jhanwar-Uniyal et al., 2019). Mitogen-induced PI3K/Akt and Ras/MEK/ERK activate the mTOR canonical and non-canonical pathways and participate in tumorigenesis, insulin resistance, osteoporosis and arthritis (Memmott and Dennis, 2009; Zou et al., 2020). A recent study has suggested that activation of mTORC1 by extracellular growth signals and intracellular LKB1 mutations alleviate the histone H2A and H2A ubiquitination following the DNA damage caused by RNF168 phosphorylation. This severely affects the DNA repair mechanism of the hepatocytes and promotes malignant cell transformation and oncogenesis (Xie et al., 2018). According to Chen et al. (2009) activated PI3K/PTEN/Akt/mTOR pathway facilitates tumour metastasis and invasion by upregulating the expression of MMP-9. Likewise, PI3K/Akt/mTOR signalling supports the survival and proliferation of colon cancer stem cells (CCSC) and plays a key role in the remission and metastasis of sporadic colon cancer (Chen et al., 2017).

## 5 Signalling cross-talks in tumour microenvironment

### 5.1 Hepatocellular carcinoma

Unresolved inflammation is the root cause of hepatic damage and the contemporaneous regeneration serves as a key propagator of hepatic fibrosis and cirrhosis (Kwon et al., 2015; Karin and Clevers, 2016). Despite the endogenous variability among numerous etiological factors, parenchymal cell death-induced inflammatory cascades and incessant wound healing remain the major implications of hepatocellular carcinoma (HCC) (Karin and Clevers, 2016). Cancer stem cells (CSCs) are responsible for tumour survival, proliferation, growth, metastasis, and remission after chemotherapy in HCC (Hanahan and Coussens, 2012). Active immune cells assist the survival and proliferation of CSCs by continuously secreting pro-inflammatory mediators that favour preneoplastic niches in TME. According to Tanimizu et al. (2017), hepatic progenitor cells exhibit malignancy when transplanted into a chronically injured liver or harvested in a similar inflammatory microenvironment. In dysplastic lesions, signs of malignancy are not apparent until the autocrine IL-6 or paracrine IL-6/STAT3 signalling is established in progenitor cells. These signalling pathways are also reported to expand the HCC-CSC phenotypes driven by TAMs (Wan et al., 2014) and propagate the hepatic progenitor cell in HBx-transgenic mice (Wang et al., 2012). The cross-talk interactions of IL-6/STAT3 and TNF-α/NF-κB by long noncoding RNA lnc-DILC further fortifies the implication of hepatic inflammation in the expansion of HCC-CSC (Wang et al., 2016). NF-κB and STAT3 have strikingly similar target genes responsible for cellular proliferation, survival, and stress-coping mechanisms. Cross-talks between these transcriptional factors were previously identified in the diethylnitrosamine (DEN)-induced liver carcinogenic injury model, and later the epidemiological data supported these signalling interactions in HCC- and prostate-related inflammation. In Kupffer cells, IL-1α released during DEN-induced injury induces IL-6 production via activating NF-κB (Maeda et al., 2005; Sakurai et al., 2008). At the same time, IL-6 activates the STAT3, upregulates target gene expression and increases the hepatocyte turnover to compensate for the cell damage. Interestingly, this hyperproliferation, which started as a coping mechanism for the damage, subverbally promotes liver tumorigenesis (Naugler et al., 2007; He et al., 2010). Additionally, activated STAT3 is also responsible for retaining the p65 in nucleus and ensuring the perpetual activation of NF-κB (Lee et al., 2009). On the contrary, dephosphorylation of activated JAK2/STAT3 by ROS-mediated oxidizing protein tyrosine phosphatases (PTPs) also governs a negative NF-κB/STAT cross-talk in HCC and plays a significant role in tumorigenesis (Sakurai et al., 2006). Aberrant methylation of suppressors of cytokine signalling (SOCS) 1 and 3 have been frequently observed in human primary HCC tumours, impeding their ability to negatively regulate cytokine signalling. Reinstating activities in cells lacking SOCS 1 and 3 expressions result in the suppression of cell growth and reversal of constitutive STAT3 phosphorylation. Conclusively, SOCS 1 and 3 methylation and its tumour growth suppression activity demonstrate the importance of the constitutive activation of the JAK2/STAT3 pathway in the development of HCC (Yoshikawa et al., 2001; Niwa et al., 2005).

### 5.2 Prostate cancer

**FIGURE 4:**
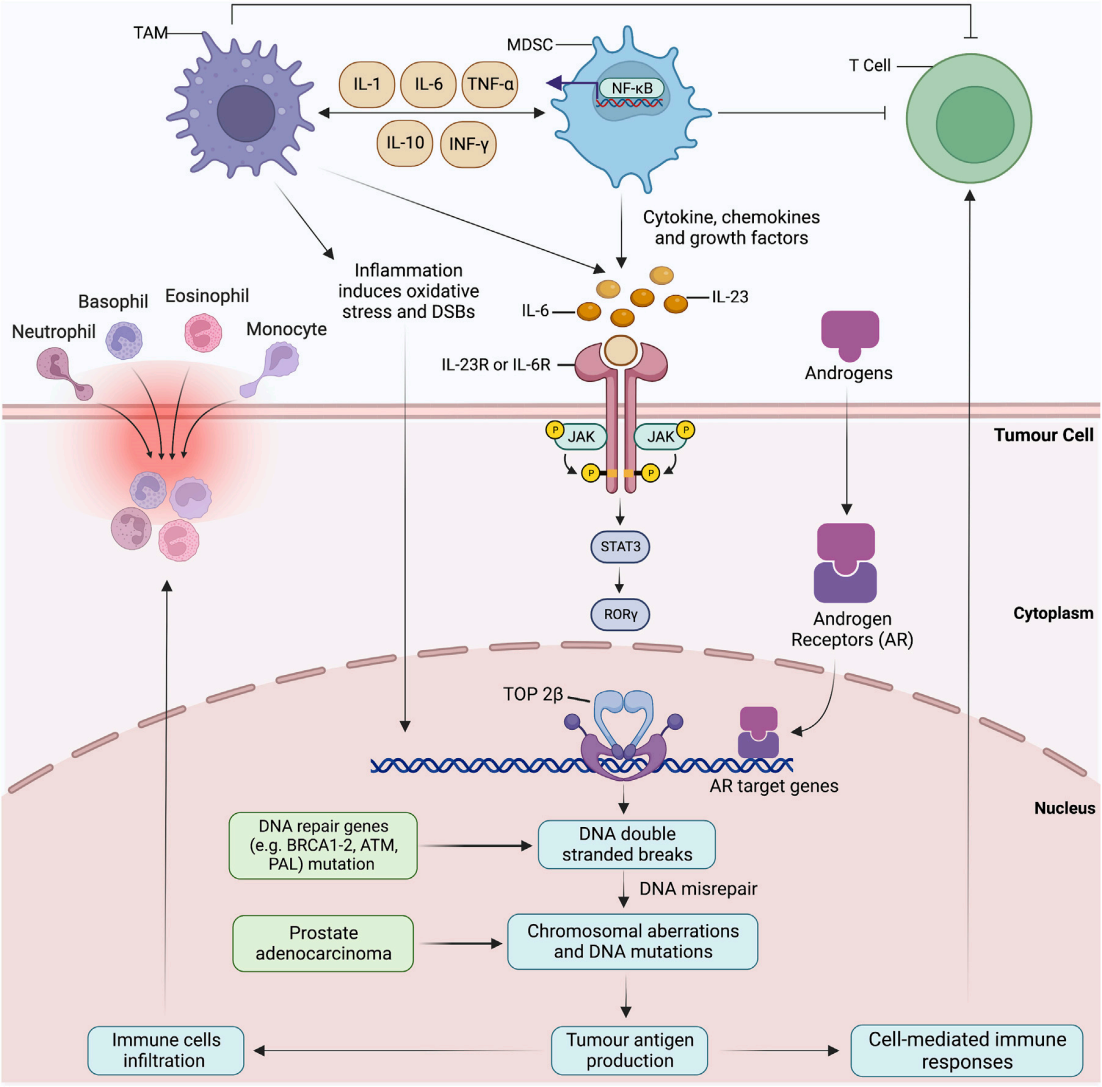
Reactive oxygen species and proinflammatory cytokines produced by tumour-associated macrophages (TAMs) and myeloid-derived suppressor cells (MDSCs) cause direct DNA damage and strand breaks. Numerous cytokines produced by TAMs and MDSCs impact the differentiation and proliferation of normal and neo-malignant cells. For instance, IL-23 produced by MDSCs prompts oxidative DNA damage, DNA mis-repairs and induce double-strand breaks (DSBs) and AR target genes by activating JAK-STAT-RORγ-mediated androgen receptor (AR) signalling. Nuclear AR signalling summons the topoisomerase 2β (TOP2B) to the promoter regions, resulting in the chromosomal co-localization of AR target genes and other transcriptional factors. Non-random rearrangements and AR-induced gene fusions occur due to the accumulation of DSBs at the target gene, mimicking the androgen-regulated TMPRSS2 to ERG. Aberrations in homologous recombination result in chromosomal proximation and sole dependency on error-prone non-homologous end-joining for DSB repairs. Malfunctioning tumour suppressor gene, AR targeted gene and DNA repair genes (BRCA1-2, ATM, PALB2, CHEK1-2) prompt the extravasation of immune cells, genome destabilisation and tumour antigen production. Inflammation-induced oxidative stress possesses the highest damage to DRGs and AR-target genes and perpetuates a never-ending loop of DNA damage and mis-repairs.

Genetic epidemiological data have suggested that germline mutations that occur during chronic inflammation significantly upregulate the risk factor of prostate cancer (Torkko et al., 2015). This genomic and cellular damage orchestrates prostate cancer by inducing somatic gene mutations responsible for tissue recovery against inflammatory storms (Torkko et al., 2015; de Bono et al., 2020). Additionally, the molecular characterisation of proliferative inflammatory atrophy-associated precursor lesions with prostate intraepithelial neoplasia and prostate cancer shares strikingly similar molecular traits (De Marzo et al., 2007). Tissue microenvironment enriched with cytokines and growth factors augments cell proliferation via supporting replication and angiogenesis (Palapattu et al., 2005; Nakai and Nonomura, 2013). Inflammatory myeloid cells, including myeloid-derived suppressor cells (MDSCs) and TAMs, are infamous for damaging and breaking DNA stands. Elevated IL-6 levels and ROS change the course of cell differentiation and function, while the malignant cells take direct advantage of these alterations (de Bono et al., 2020). MDSCs-driven IL-23 production activates the JAK-STAT-RORγ-mediated androgen receptor (AR) signals. Upon activation, nuclear AR signalling summons the topoisomerase 2β (TOP2B) to the promoter regions of AR target genes (Calcinotto et al., 2018). The recruitment of TOP2B enzymatically hastens the double-strand breaks (DSBs), resulting in DNA mis repairs and creating a disparity between error-prone non-homologous end-joining and homologous recombination (Figure 4). As the DSBs start congregating at the target gene, non-random rearrangements and fusion occur. Altogether, these events propagate the extravasation of immune cells, genetic instability and tumour antigen production (Lin et al., 2009; Haffner et al., 2010). Chronic inflammation is a driving force for DNA damage, but transcriptionally active AR targets and DNA repair genes bear the highest damage tool. Numerous studies have supported the IL-6 role in prostate cancer by reporting upregulated IL-6R expression and elevated IL-6 levels in prostatic intraepithelial neoplasia (PIN) and hormone-refractory prostate cancer (Ouyang et al., 2005; Markowski et al., 2008). These elevated levels also serve as a principal activating factor for AR and inducible enzymes such as COX-2. The latter is overexpressed predominately in proliferative inflammatory atrophic (PIA) regions, a predicted risk factor lesion to prostate cancer (Shinohara et al., 2013; Ashok et al., 2019). Lastly, IL-6 promotes the epigenetic transformed states by establishing cross-talk interaction between STAT3 and NF-κB.

### 5.3 Colorectal cancer

Chronic ulcerative colitis (UC) and Crohn’s disease (CD) are two major divisions of inflammatory bowel disease (IBD). These progressive inflammatory conditions have the potential to further transform into colorectal cancer (CRC) (Long et al., 2017). Of all CRC etiological factors, chronic inflammation and elevated epithelial turnover remain the most significant due to their putative role in dysplastic precursor lesion development (Ullman and Itzkowitz, 2011). Activated immune cells pose direct DNA damage by producing genotoxic ROS and reactive nitrogen intermediates (RNI) in epithelial cells. Chronic inflammation-induced perforations allow the food-borne mutagens to breach the protective intestinal barriers and interact with inflamed epithelium (Long et al., 2017). Proinflammatory cytokines such as IL-1 and IL-6 induce chromatin modifiers, upregulate miRNA and pose epigenetic changes by activating JAK/STAT3 and NF-κB (Ding et al., 2019). In return, these transcriptional factors increase the turnover of mediators involved in angiogenesis (VEGF, IL-8, PGE_2_), metastasis (MMP9) and proliferation (CyclinD1) by establishing cross-talk interactions between COX2/PGE_2_, PI3K-AKT, ERK and Wnt-β-catenin (Wang et al., 2009; Oshima et al., 2011; Long et al., 2017). The cross-talk begins with Wnt/β-catenin that, upregulates the Notch expression and triggers the Jagged-1 activation (Bertrand et al., 2012). Notch simulates the EGRF pathways in colonic carcinogenesis and simultaneously activates PI3K/AKT and MAPK/ERK pathways (Staudacher et al., 2017). Transcription factor hairy and enhancer of split-1(HES-1) with direct inhibitory effect on phosphatase and tensin homolog (PTEN) activates PI3/AKT pathway (Rajendran et al., 2017). Additionally, PI3K and β-catenin suppress glycogen synthase kinase 3 beta (GSK3β) and APCAxin/GSKb3 complexes along with their downstream target genes that regulate the activities of suppressor of mothers against decapentaplegic (SMADs). Lastly, Notch signalling upregulate anti-apoptotic gene expression of Bcl-2, Bcl-XL, XIAP and survivin (Hristova et al., 2013). According to Wu et al. (2013) cross-talk of PI3K/Akt, MAPK, Notch and Wnt/β-catenin is reported to regulate survivin and XIAPs in a positive manner. TGF-β signalling is said to downregulate the anti-apoptotic gene expressions (Wu et al., 2013). Collectively, these events allow the epithelial cells to circumvent the cell cycle checkpoint, evade apoptosis and proliferate without check and balance in CRC (Bertrand et al., 2012).

### 5.4 Squamous and non-squamous cell lung carcinoma

Chronic bronchitis (CB) refers to airway inflammation that leads to mucus production, shortness of breath, dyspnoea, wheezing and chest tightness. Nevertheless, the differential diagnosis of CB is difficult since these symptoms overlap with chronic obstructive pulmonary disease (COPD) (Durham and Adcock, 2015; Park et al., 2020). The key association between chronic bronchitis and pulmonary cancer lies in the hyperactivation of NF-κB by ROS and pro-inflammatory cytokines (Karin, 2009; Liu et al., 2017). These hyperactivated states are frequently reported in the inflammatory cells of lower airway bronchial epithelium in CB, premalignant lesions of the pulmonary epithelium and neoplastic cells of squamous cell lung carcinoma (Sheller et al., 2009). Chronic bronchitis and squamous cell lung carcinoma (SCLC) are cross-linked with NF-κB-associated transactivation of inflammation-related genes (Zaynagetdinov et al., 2016; Rasmi et al., 2020). COPD patients with a long history of smoking are at the highest risk. Cigarette smoke promotes NF-κB-dependent inflammatory response and establishes interactions with other signalling pathways, including peroxisome proliferator-activated receptors (PPARs), PI3K/AKT, p38 MAPK and JAK/STAT. These cross-talk interactions with the continuous assistance of IL-6 give rise to anti-apoptotic activity, poor differentiation and drug resistance in SCLC (Huang W.-L. et al., 2010; Adcock et al., 2011). The late study of Yoshida et al. (2008) reported that chronic nicotine exposure increases acetylcholine (ACh) production and cholinergic receptor (AChR) activity, which are reported as key cell proliferation enhancers in non-squamous cell lung carcinoma. Moreover, excessive ACh release facilitates tumour angiogenesis by increasing the production of hypoxia-inducible factor (HIF)-1 and VEGF (Zhang et al., 2007; Rooney and Sethi, 2011). Additionally, by establishing cross-talks with MAPK and NF-κB, activated nicotine receptors upregulate the activity of gene responsible for survival in neoplastic cells (Carlisle et al., 2007). Nevertheless, late studies of Rooney and Sethi (2011) and Zhang et al. (2007) suggested that darifenacin M3 mAChR antagonist activity has the potential to abrogate the AChR-driven cell proliferation and growth via abolishing AChR-MAPK cross-talk in SCLC.

Hypermethylation of SOCS 3 promoter is known for initiating the cross-talks between NF-κB and JAK/STAT in numerous lung cancers. Erythropoietin receptor pathways, activated nicotinic receptors and EGFR are potential activators of STAT3 (He et al., 2004; Lai and Johnson, 2010). Nevertheless, IL-6 plays a pivotal role in activating STAT3 and establishing the interaction between PI3K/Akt and NF-κB. Activated STAT3 enters the perpetuating cycle of IL-6 autocrine production. It creates a positive feedback loop with the JAK/STAT activation, thus promoting progression and chemotherapy resistance in lung cancers (Huang W.-L. et al., 2010). It is worth mentioning that therapeutic strategies based on JAK/STAT inhibition have shown success in promoting apoptosis and anti-proliferative activities in SCC, suggesting that these pathways are the potential target sites for the treatment of lung cancers, including NSCLC and SCLC (He et al., 2004).

Peroxisome proliferator-activated receptors (PPARs) belong to the nuclear hormone receptor superfamily (Afzal et al., 2020). These ligand-activated transcription factors bind with the promoter region of the targeted gene at PPAR specific sequence of response elements (Afzal et al., 2021; Afzal et al., 2022). Other than metabolism, PPAR has a putative role in cell differentiation and apoptosis via mediating the activities of transcriptional factors, including NF-κB. According to Belvisi and Mitchell (2009), proinflammatory cytokine from the airway epithelium and epithelial cell differentiation are regulated by PPAR ligands. For instance, PPAR agonist rosiglitazone is reported to increase the neutrophil count and suppress chemoattractant production in lipopolysaccharide-induced COPD-like airway inflammatory animal models (Belvisi and Mitchell, 2009). Hence, it is unsurprising that downregulated PPAR expression has been frequently observed in numerous lung cancers, including SCC and NSCLC (Li et al., 2010; Adcock et al., 2011).

### 5.5 Head and neck squamous cell carcinoma

Head and neck squamous cell carcinoma (HNSCC) is derived from the oral cavity, larynx and pharynx mucosal epithelium. Frequent consumption of tobacco, alcohol abuse, human papillomavirus (HPV) infection and chronic mucosal epithelial inflammation is generally associated with HNSCC (Johnson et al., 2020; Esteban et al., 2021). Upregulated EGFR expression and activation of EGFR-mediated STAT3, RAS-ERK and PI3K-AKT pathways are frequently reported in HNSCC (Amornphimoltham et al., 2004; Geiger et al., 2016). Nevertheless, few studies have supported the EGFR-independent activation of the said signalling pathways (Sriuranpong et al., 2003; Amornphimoltham et al., 2005). The activation of STAT3-dependent genes starts with tyrosine705 phosphorylation of STAT3 by EGFR. Sriuranpong et al. (2003) suggested that the rapid tyrosine phosphorylation followed by the STAT3-DNA complex formation is unaffected by the EGFR inhibition in HNSCC cell lines. Of interest, the proposed EGFR-independent STAT3 mechanism involves the activation of the gp130 cytokine receptor subunit, which propagates the tyrosine705 phosphorylation via activating the intracellular JAK tyrosine kinases. It is worth mentioning that IL-6 secreted in HNSCC serves as a key activator of gp130 by binding with the IL-6 receptor-gp130 complex present on the surface of HNSCC cells. While few other studies have proposed that dysregulated EGFR activity or tumour-released cytokines in an EGFR-independent fashion are mainly responsible for the perpetual activation of STAT3 in HNSCC (Siavash et al., 2004). These findings, together with the published report of Squarize et al. (2006) suggested that upregulated expression of IL-6 increases the transcription by involving IL-6 promoter. However, the presence of intact NF-κB response elements of IL-6 transcription initiation site is a prerequisite for optimal IL-6 promoter functioning. In the same study, NF-κB inhibition decreased IL-6 production, downregulated its expression and suppressed the secretion of IL-8, IL-10, GM-CSF and G-CSF. Furthermore, STAT3 reciprocates to NF-κB inhibition by decreasing the tyrosine705 phosphorylation, interfering with the STAT3 activation in nontumorigenic epithelial cells in a paracrine manner. The proposed cross-talk interaction of NF-κB and STAT3 pathways driven by IL-6 release and IL-6 promoter activation serves as an excellent example that the aberration of multiple interrelated signalling pathways, instead of only one deregulated biochemical route, contributes to the squamous cell carcinogenesis.

### 5.6 Ovarian cancer

Pathogenic infections are the major cause of pelvic inflammatory disease (PID) that pose harmful effects on female reproductive organs, including the cervix, fallopian tubes and ovaries (Brunham et al., 2015). Mounting evidences have suggested a strong correlation of PID with increasing incidence of ovarian cancer (OvCa) (Rasmussen et al., 2013; Rasmussen et al., 2017). The cross-talk PI3K and NF-κB have been implicated in the decease survival rate of OvCa patients. Activation of NF-κB takes place by interacting with PI3K p110a and p85 regulatory subunits (Sizemore et al., 1999; Koul et al., 2001). Upregulated p110α expression directly activates the p65/RelA by phosphorylating the IKK complex and promoting nuclear translocation. Activated PI3K-dependent AKT phosphorylation further activates the p65/RelA subunit. At this point, phospho-AKT driven IKKα phosphorylation paves the pathway for the concurrent IkB phosphorylation, which further promotes the NF-κB nuclear translocation (Takada et al., 2007). Nevertheless, Madrid et al. (2001) proposed an alternative IKK-independent pathway, which involves AKT-induced NF-κB activation and phosphorylation of the p65/RelA subunit. It is worth mentioning that cross-talk of PIK3CA, AKT1/2/3 and NF-κB subunits have also been repeatedly highlighted in the data analysis of Cancer Genome Atlas (Ghoneum and Said, 2019). Based on the premise, therapeutic strategies targeting the PI3K/AKT/mTOR/NF-κB axis have shown promising results against OvCa in pre-clinical models. Recently Wei et al. (2019) suggested that PI3K/AKT/mTOR regulation with YAP inhibitor peptide 17 is reported to suppress the malignancy and progression of OvCa. Likewise, OvCa cell survival and cisplatin resistance are reported to be significantly suppressed with the PI3K and ghrelin receptor blockade with [D-Lys3]-GHRP-6 and LY294002, respectively. On the contrary, activation of PI3K/AKT/mTOR/NF-κB axis with growth hormone secretagogue receptor (GHSR) ligand ghrelin is suggested to augment cell survival and augment cisplatin resistance in OvCa (El-Kott et al., 2019).

## 6 Cross-talk interaction targeted therapies; friend and foe

### 6.1 Therapeutic opportunities

Over the past few decades, cancer treatments have been designed to target specific signalling pathways involved in cell survival and proliferation. Henceforth, the usage of non-selective cytotoxic drugs has been significantly reduced with the emergence of targeted therapies focusing on specific molecular pathways with enhanced biochemical and cancer selectivity. Tumour-driven mutations are easier to identify with next-generation DNA sequencing, making targeted therapy effective and the development of precision medicine expeditious (The Cancer Genome Atlas Research Network, 2008; Alifrangis and McDermott, 2014). For inflammation-associated cancers, targeted therapies focusing on the signalling pathways and gene mutations responsible for feedback regulations have shown promising therapeutic results. However, it is anticipated that the effectiveness of drug therapies varies, and drug resistance arises due to tumour genomic variability, intralesional heterogeneity and cross-talk between signalling pathways (Prahallad and Bernards, 2016).

Alternatively, these cross-talks can be used strategically as a valuable tool to promote synthetic lethality, in which inhibition of two individual pathways (gene) shows no fatal response, but simultaneous inactivation of two pathways exhibits significant lethality (Ashworth, 2008). For instance, in the presence of a mutated cancer signalling pathway, the inactivation of a second pathway bearing a cancer-specific defect will theocratically be lethal to the cancer cells. The clinical application of this concept was established by Rehman et al. (2010) and colleagues with a conscientious observation that mutant BRCA1 is highly sensitive to poly ADP-ribose polymerase (PARP) inhibitors. Also, if a substitute pathway is available to repair damaged DNA, BRCA1 mutant tumours will tolerate homologous recombination defects. However, when PARP 1 and 2, key enzymes of the alternative pathways involved in double-stranded DNA repair are blocked with PARP inhibitor, it will result in the instantaneous BRCA1 mutant tumour cell death. Similarly, in colon cancer, the categorical suppression of all kinases results in EGFR attenuation, implicated as synthetic lethal with BRAF(V600E) inhibition. Of interest, ERK and the PI3K signalling pathways are reactivated due to the BRAF(V600E) inhibition which paves the way for EGFR feedback activation (Corcoran et al., 2012; Prahallad et al., 2012). The approval of PARP inhibitor Olaparib for BRCA-mutated ovarian cancer treatment has substantiated the future clinical applications of this synthetic lethality.

An alternative coping strategy is to address tumour and inflammatory microenvironment collectively. With this approach, complex signalling communication between tumour-infiltrating immune cells responsible for angiogenesis, proliferation and metastasis becomes addressable altogether. Numerous studies have already been discussed, which highlighted the intricate interactions between resident macrophages, IL-6, COX-2/PGE_2_ and CXCL12 in the breast cancer microenvironment (Garcia-Tuñón et al., 2005; Martinet et al., 2010; Chow and Luster, 2014). Moreover, aberrant transcriptional activities of MAPK and NF-κB upregulate the expression of growth-stimulating genes. Regarding vascularisation, CXCL12 and CCL2 are the most potent angiogenic chemokine, which enables the adequate oxygen supply required in metastatic breast cancer. Moreover, CCL2 release from mammary tumours attracts the CCR2-expressing monocytes produced by bone marrow. According to Bonapace et al. (2014) and team, upregulated expression of CCR2 is correlated with decease chemotherapy sensitivity and poor clinical outcomes. In their study findings, an anti-CCL2-neutralising antibody was an effective treatment in obstructing the release of monocytes and abrogating metastasis. In contrast, few studies have implicated TME in decreasing cancer cells’ sensitivity towards targeted or conventional chemotherapies (Straussman et al., 2012). Based on the premise, it is established that the effectiveness of the target therapies is affected and governed by the complex interactions of epithelial tumours and their undisciplined microenvironment.

### 6.2 Therapeutic restraints

The drug combinations seem to be an optimistic approach to block core or multiple signalling pathways (cross-talk) and improve clinical outcomes. Practically, combinations of targeted therapies are far from ideal when factors like secondary gatekeeper mutations, toxicity and tolerability are to be dealt with frequently in clinical settings. In the current scenario, there are three main strategies for combining target agents; multiple drugs aiming same target site (trastuzumab and pertuzumab to target HER2 in breast cancer), aiming multiple target sites on the same pathway (AKT and MEK inhibitors in HCC) and using multiple targeted drug combinations simultaneously aiming numerous cellular mechanisms (alkylating agents promoting cell cycle progression and pro-apoptotic activities) (Dancey and Chen, 2006; Kummar et al., 2010). Nevertheless, the lack of comprehensive knowledge regarding the intricacy of signalling pathways considerably undermined the risk-to-benefit ratio of the proposed strategies. To fill this knowledge gap, these combination strategies were tested against four different types of cancers, including solid tumours, melanoma, glioma and renal cell cancer, in pilot clinical trials (Kummar et al., 2010). The interim analyses of these trials have exhibited promising clinical efficacy for some combinations but not for others, suggesting the empirical nature of this mix-and-match combination process. For instance, the mixture of multi-kinase inhibitors sorafenib and bevacizumab showed some promising results in ovarian cancer and renal cell carcinoma treatment, but the clinical trial was terminated earlier due to dose-limiting toxicities associated with drug combination therapy (Azad et al., 2008; Sosman, 2008). Similarly, when sorafenib and temsirolimus (mTOR inhibitor) combinations were explored for glioblastoma multiforme management, the development of grade 3 thrombocytopenia at the maximum tolerated dose led to the termination of the trial (Lee et al., 2012). It is evident from the above-stated studies that any clinical trial with a lack of understanding of signalling cross-talk interactions and molecular rationale will have a limited chance of success.

## 7 Concluding remarks

Over the past few decades, the conceptualisation of inflammation-induced cancers and the functional relationship between inflammation and cancer has been well recognised. The key orchestrators of the signalling pathways that drive the molecular events and establish the cross-talk interactions between stromal, tumour and inflammatory immune cells in the microenvironment have been firmly established. Of interest, we have gasped a vast array of knowledge on the resident cells and signalling pathways involved in cancer-promoting inflammation, intrinsic and extrinsic (inflammatory) pathways, tumour suppressor gene dysfunction, neoplasm, metastasis, and prognosis. Despite these establishments, key molecular mechanisms that connect the immune-modulatory effects of inflammation with the various stages of cancer development remain elusive. Elucidation of these molecular gateways is decidedly warranted because they hold the answer key to the fundamental questions that incapacitate our absolute knowledge about cancer-related inflammation. For instance, if chronic inflammation eternally promotes pro-tumour activity, then as Nickoloff et al. (2005), why does inflammation defiantly decrease cancer in chronic inflammatory diseases such as psoriasis? Moreover, how come the late study of Mantovani et al. (1992) reported that densely populated microenvironments with eosinophil and TAMs lead to better prognosis in colon and breast tumours? These erratic observations are concerning but should not be casually ignored since these counterintuitive studies challenge our limited knowledge and force us to revisit our oversimplified explanations of cancer-promoting inflammatory processes.

The cross-talks of signalling pathways, developed initially to support homeostasis during inflammatory processes, change their role and promote atypical proliferation, survival, angiogenesis, and subversion of adaptive immunity in TME. These transcriptional and regulatory pathways invariably contribute to cancer-promoting inflammation in chronic inflammatory disorders and foster smouldering inflammation in the microenvironment of various tumour types like breast tumours (Balkwill et al., 2005). Besides the identification of common target sites of numerous cancers, these signalling programs and cross-talks governing immune cells’ plasticity and functional diversity can be used to develop new fate-mapping lineage-tracing mechanisms.

Cancer precision therapies targeting signalling pathways and corresponding genetic mutation involved in feedback regulations have shown promising future prospects. Nevertheless, devising drug combinations to cater for neoplasm, secondary gatekeeper mutations, and toxicity by conventional “trial and error” testing has shown marginal success. One effective strategy to develop a powerful drug combination is synthetic lethality genetic screening, but this approach has its demerits. Firstly, investigational studies do not consider the clinical effectiveness of proposed synthetic lethal interactions, and their acknowledgements were merely context-driven (Barbie et al., 2009; Luo et al., 2009; Singh et al., 2012). Secondly, it is challenging to foresee synthetic lethal drug interactions if cancer genotype is a decisive factor in targeted therapy selection. Considering these scepticisms, the first drug using the concept of synthetic lethal has been approved, paving the way for many others to follow. Lastly, we need to completely reassess the practice of qualifying a drug for combination therapy based on its single-agent activity. In their recent study, Rudalska et al. (2014) and colleagues skilfully demonstrated this unorthodox paradigm by employing the concept of synthetic lethal interaction. They successfully transformed MAKP14 inhibitors with poor anticancer activity (as a single agent) into a strong chemotherapeutic candidate by adding these drugs in combination with sorafenib.
